# Clinical characteristics, disability outcomes, and disease-modifying therapy patterns in late-onset multiple sclerosis: a single-center retrospective cohort study

**DOI:** 10.3389/fneur.2026.1966162

**Published:** 2026-10-21

**Authors:** Ruslan Alomar, Elīna Polunosika, Guntis Karelis, Daina Pastare

**Affiliations:** 1 Faculty of Medicine, Rīga Stradin´š University, Riga, Latvia; 2 Neurology and Neurosurgery Department, Riga East University Hospital, Riga, Latvia; 3 Neurology and Neurosurgery Department, Rīga Stradin´š University, Riga, Latvia

**Keywords:** anti-CD20 therapy, disability, disease-modifying therapy, expanded disability status scale, late-onset multiple sclerosis, magnetic resonance imaging

## Abstract

**Background:**

Late-onset multiple sclerosis (LOMS), defined here as symptom onset at age 50 years or later, is associated with diagnostic complexity, a higher prevalence of progressive phenotypes, greater disability, and challenging disease-modifying therapy (DMT) decisions. We characterized clinical manifestations, magnetic resonance imaging (MRI) lesion distribution, disability outcomes, and DMT patterns in a Latvian tertiary-care cohort.

**Methods:**

We retrospectively reviewed medical records from Riga East University Hospital. Of 518 patients in the source multiple sclerosis dataset, 59 met the LOMS definition; after exclusion of two patients with clinically isolated syndrome and one with an unspecified phenotype, 56 patients with relapsing-remitting, primary progressive, or secondary progressive multiple sclerosis were analyzed. Clinical symptoms, MRI lesion localization, baseline and five-year Expanded Disability Status Scale (EDSS) scores, EDSS change, and initial and current DMT patterns were assessed using nonparametric and exact tests.

**Results:**

The cohort included 38 women and 18 men; 38 patients had relapsing-remitting multiple sclerosis and 18 had progressive multiple sclerosis. Progressive phenotypes were associated with more frequent pyramidal/motor symptoms (100.0% vs. 76.3%; *p* = 0.045) and bowel/bladder dysfunction (72.2% vs. 42.1%; *p* = 0.035). Baseline EDSS was higher in progressive disease (median 3.75 vs. 2.00; *p* < 0.001), as was five-year EDSS among the 39 patients with available follow-up (median 6.00 vs. 3.00; *p* < 0.001), whereas EDSS change did not differ significantly (*p* = 0.377). Anti-CD20 and other high-efficacy DMTs were used more frequently in progressive phenotypes and in patients with greater observed disability.

**Conclusion:**

Progressive LOMS phenotypes were associated with greater motor and sphincter symptom burden and higher disability, but the absence of a difference in unadjusted EDSS change should not be interpreted as equivalent disability progression. Treatment associations primarily describe real-world selection and are subject to confounding by indication.

## 1 Introduction

Multiple sclerosis (MS) is a chronic immune-mediated disease of the central nervous system characterized by inflammation, demyelination, and neurodegeneration. Although MS most often begins in young adulthood, clinical onset may occur after 50 years of age. This subgroup is generally defined as late-onset multiple sclerosis (LOMS), with most contemporary studies using symptom onset at 50 years or older as the defining threshold (1–3, 13).

LOMS is clinically important because it is associated with distinct clinical characteristics and a more complex diagnostic and therapeutic context. Systematic reviews and registry studies have reported that LOMS accounts for approximately 5% of all MS cases, although the reported proportion varies by population, diagnostic criteria, and data source (1). In a Swedish nationwide study, LOMS represented 11.8% of included MS patients and was associated with a higher proportion of primary progressive MS and a greater risk of reaching disability milestones (3).

The diagnosis of MS in older patients may be difficult because age-related vascular white matter lesions, degenerative spinal disease, vitamin B12 deficiency, vasculopathies, infections, neoplasms, neuromyelitis optica spectrum disorder, MOG antibody-associated disease, and other mimics are more frequent in this age group (4, 5). Radiological and cerebrospinal fluid findings, particularly spinal cord magnetic resonance imaging (MRI) and cerebrospinal fluid oligoclonal bands, may be especially valuable when applying diagnostic criteria in older-onset patients (6, 18).

Therapeutic decision-making is also more challenging in LOMS. Older adults with MS have historically been underrepresented in pivotal DMT trials, while immunosenescence, comorbidities, polypharmacy, infection risk, and reduced inflammatory activity may alter the benefit-risk profile of DMTs (7, 8). At the same time, recent real-world evidence suggests that chronological age alone should not be used to exclude selected older patients from DMT consideration when inflammatory activity is present (9).

The clinical profile of late-onset multiple sclerosis is not a newly recognized phenomenon. Historical studies, including the work of Cazzullo et al. (10) described a predominance of motor manifestations, progressive disease course, and greater disability among patients with later disease onset. Nevertheless, important questions remain regarding whether these historical observations are consistently reproduced across contemporary healthcare settings and how LOMS is currently diagnosed, monitored, and treated in the era of MRI-based diagnostic criteria and high-efficacy disease-modifying therapies.

Published full-length evidence on LOMS in Latvia remains very limited. Country-specific real-world data are valuable not because they replace larger international cohorts, but because they test the external validity of established observations and document how contemporary diagnostic and therapeutic strategies are applied in different clinical settings. To our knowledge, no previous full-length Latvian study has jointly evaluated phenotype-specific clinical manifestations, MRI lesion distribution, longitudinal EDSS outcomes, and initial-to-current DMT patterns in patients with MS symptom onset at age 50 years or later.

Therefore, the objective of this study was not to redefine the established LOMS phenotype, but to examine its expression in a contemporary Latvian tertiary care cohort and to extend previous evidence through an integrated assessment of clinical manifestations, MRI lesion localization, baseline and five-year disability, and real-world DMT patterns. The primary objective was to compare baseline and five-year EDSS outcomes between RRMS and progressive MS phenotypes. Secondary exploratory objectives were to describe symptom profiles, MRI lesion localization, and initial and current DMT patterns.

## 2 Materials and methods

### 2.1 Study design and setting

This single-center retrospective cohort study was based on the medical records of patients with LOMS followed at Riga East University Hospital, Latvia. Clinical data collected between 2014 and 2024 were retrospectively reviewed. The study is reported in accordance with the STROBE statement and the RECORD extension for observational studies using routinely collected health data.

### 2.2 Study population

Following data extraction, duplicate records were identified using unique patient identifiers and removed before creation of the source dataset. Missing values were retained as missing and were not imputed. The resulting source dataset comprised 518 unique individuals. MS diagnoses were established by treating neurologists according to the diagnostic criteria applicable at the time of diagnosis and remained clinically confirmed during subsequent specialist MS follow-up (17).

Patients were eligible for inclusion if they met all of the following criteria: confirmed MS diagnosis, symptom onset at ≥50 years of age, available medical documentation on disease course and MS phenotype, and available data on EDSS assessment, MRI findings, and disease-modifying treatment. The initial LOMS working dataset included 59 patients, corresponding to 11.4% of the source MS dataset. The study sample size was determined by the number of eligible LOMS cases available in the source dataset during the study period.

Patients were excluded from the main comparative analysis if they were classified as clinically isolated syndrome (CIS; *n* = 2) or had an unspecified MS phenotype (*n* = 1), because these categories did not align with the predefined phenotype-based comparisons. Patients with insufficient documentation to determine MS phenotype or key outcome variables were also excluded from phenotype-based analyses. The final analytic cohort included 56 patients with RRMS, PPMS, or SPMS, corresponding to 10.8% of the initial source MS dataset.

### 2.3 Variables and definitions

The following variables were extracted: patient identifier, sex, current age, age at MS onset, MS phenotype, clinical symptoms, MRI lesion localization, comorbidities, current and initial DMT, baseline EDSS, EDSS after five years, and EDSS delta, defined as five-year EDSS minus baseline EDSS. MS phenotypes were coded as relapsing-remitting MS (RRMS), primary progressive MS (PPMS), and secondary progressive MS (SPMS). For two-group comparisons, PPMS and SPMS were combined into a progressive MS group. Baseline EDSS was defined as the earliest EDSS score documented in the available medical record, which in most cases was recorded at or around the time of MS diagnosis.

Clinical symptoms were coded as binary variables and included sensory symptoms, visual symptoms, pyramidal/motor symptoms, bowel and bladder dysfunction, cerebellar/balance symptoms, cognitive symptoms, headache, cerebral symptoms, pain, and brainstem symptoms. MRI lesion distribution was categorized as periventricular, infratentorial, juxtacortical, optic nerve involvement, and spinal cord. MRI lesion localization was extracted from routine neuroradiology reports available in the medical records. MRI lesion localization was extracted from routine neuroradiology reports in the longitudinal medical record. Brain, spinal cord, and optic nerve MRI data were available for all 56 patients. For each anatomical region, a value of 0 indicated that no lesion was identified in that region; therefore, the full analytic cohort (*n* = 56) was used as the denominator for MRI percentages.

### 2.4 Treatment classification

Initial and current DMTs were analyzed using the same treatment classifications. First, exact individual therapies were summarized descriptively. Second, active DMT users were grouped as receiving anti-CD20 therapy (ofatumumab or ocrelizumab) vs. other active DMTs. Patients without DMT and those receiving prednisolone only were excluded from this active-DMT comparison. Third, treatment intensity was grouped as no DMT/steroid only, platform/moderate DMT, or high-efficacy DMT. Platform/moderate DMT included glatiramer acetate, dimethyl fumarate, teriflunomide, ponesimod, interferon beta-1a, peginterferon beta-1a, diroximel fumarate and mitoxantrone. These study-specific categories were used to describe treatment patterns rather than to evaluate treatment efficacy.

### 2.5 Outcomes

The main disability outcomes were baseline EDSS, EDSS after five years, and EDSS delta. Five-year EDSS was analyzed in patients with an available five-year EDSS value in the clinical record. For the sensitivity analysis, patients with an age-derived disease duration of less than five years (current age minus age at symptom onset, using the recorded whole-year ages) were excluded and the EDSS comparisons were repeated.

### 2.6 Statistical analysis

Statistical analysis was performed using IBM SPSS Statistics (IBM Corp., Armonk, NY, United States), version 31.0.1.0 (build 49). Continuous and ordinal variables were summarized as medians and interquartile ranges (IQRs). Categorical variables were summarized as counts and percentages. Because EDSS is an ordinal scale and the sample size was limited, Mann–Whitney *U* tests were used for two-group comparisons and Kruskal–Wallis tests for comparisons involving three groups. Categorical variables were compared using Pearson chi-square tests, Fisher exact tests, or Fisher–Freeman–Halton exact tests when expected cell counts were small. Pairwise comparisons were adjusted using Bonferroni correction. A *p*-value < 0.05 was considered statistically significant.

For graphical presentation of binary symptom proportions, 95% Wilson confidence intervals were calculated.

### 2.7 Ethical considerations

The study was approved by the Research Ethics Committee of Riga Stradinš University, Latvia (approval No. 2-PEK-4/616/2025; decision date: 8 April 2025). Institutional permission was obtained from the Science Department of Riga East University Hospital (permission No. AP/08-08.1/25/103; valid until 31 August 2027). Because of the retrospective study design and the use of anonymized routinely collected clinical data, the requirement for written informed consent was waived. All data were anonymized and analyzed in accordance with applicable data protection and confidentiality regulations.

## 3 Results

### 3.1 Study cohort

Of the 59 initially identified patients, 56 met the eligibility criteria and were included in the final analytic cohort (Figure 1). The cohort included 38 women (67.9%) and 18 men (32.1%). The median current age was 62.0 years (IQR 58.8–66.2), and the median age at symptom onset was 56.0 years (IQR 52.8–59.0). RRMS was present in 38 patients (67.9%), PPMS in 9 patients (16.1%), and SPMS in 9 patients (16.1%). For comparative analyses, PPMS and SPMS were combined into a progressive MS group comprising 18 patients (32.1%). Baseline demographic and clinical characteristics are presented in Table 1.

**Figure 1:**
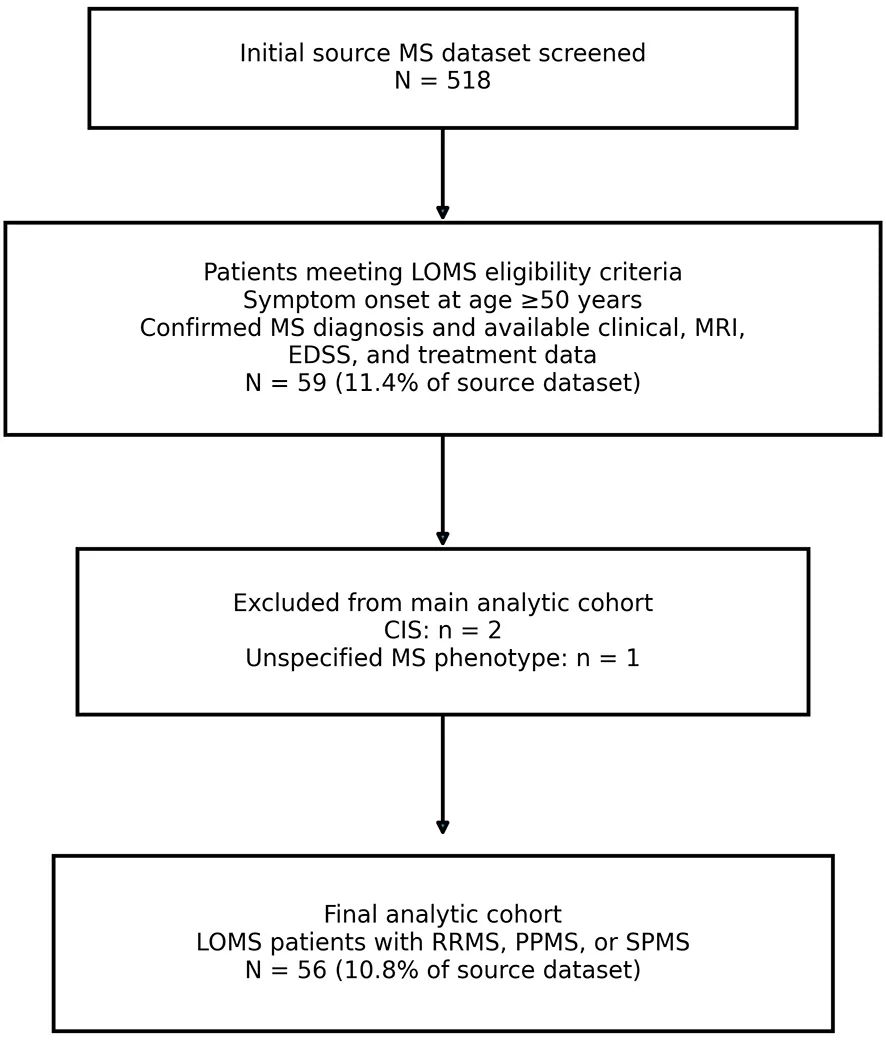
Patient inclusion flow diagram for the final analytic cohort.

**Table 1:** Baseline characteristics and EDSS outcomes by MS phenotype group.

Characteristic	Overall (*n* = 56)	RRMS (*n* = 38)	Progressive MS (*n* = 18)
Female sex	38 (67.9%)	25 (65.8%)	13 (72.2%)
Male sex	18 (32.1%)	13 (34.2%)	5 (27.8%)
Current age, years, median (IQR)	62.0 (58.8–66.2)	61.0 (57.2–65.0)	62.5 (61.0–67.8)
Age at onset, years, median (IQR)	56.0 (52.8–59.0)	56.0 (52.2–59.0)	55.5 (53.0–59.5)
Disease duration, years, median (IQR)	6.0 (3.0–9.0)	5.5 (2.2–7.0)	8.5 (4.2–11.0)
Baseline EDSS, median (IQR)	2.5 (2.0–4.1)	2.0 (1.5–3.0)	3.8 (3.1–5.9)
EDSS delta, median (IQR)	0.5 (0.0–1.5)	0.5 (0.0–1.5)	1.0 (0.0–1.5)
Five-year EDSS available, n (%)	39 (69.6%)	25 (65.8%)	14 (77.8%)
Five-year EDSS, median (IQR)	3.5 (2.5–6.0)	3.0 (2.0–4.0)	6.0 (4.8–6.4)

Values are *n* (%) or median (IQR). Progressive MS includes PPMS and SPMS. Five-year EDSS was available for 25 RRMS and 14 progressive MS patients (39 total).

EDSS, Expanded Disability Status Scale; IQR, interquartile range.

### 3.2 Clinical symptoms and MRI lesion localization

Based on the earliest documented clinical presentation in the medical records, the most frequently reported symptoms were pyramidal/motor symptoms (83.9%), sensory symptoms (71.4%), cerebellar/balance symptoms (71.4%), and brainstem symptoms (62.5%). Progressive MS showed a higher frequency of pyramidal/motor symptoms than RRMS (100.0% vs. 76.3%; Fisher's exact *p* = 0.045), and bowel and bladder dysfunction was also more frequent in progressive MS (72.2% vs. 42.1%; Pearson chi-square *p* = 0.035; Fisher's exact *p* = 0.047). Cerebral symptoms were recorded only in the RRMS group (21.1% vs. 0.0%; Fisher's exact *p* = 0.044); however, this finding should be interpreted with caution because it was based on only eight patients, all of whom had RRMS (Table 2; Figure 2).

**Table 2:** Clinical symptom profile by MS phenotype group.

Symptom	Overall (*n* = 56)	RRMS (*n* = 38)	Progressive MS (*n* = 18)
Sensory symptoms	40 (71.4%)	30 (78.9%)	10 (55.6%)
Visual symptoms	26 (46.4%)	20 (52.6%)	6 (33.3%)
Pyramidal/motor symptoms	47 (83.9%)	29 (76.3%)	18 (100.0%)
Bowel/bladder symptoms	29 (51.8%)	16 (42.1%)	13 (72.2%)
Cerebellar/balance symptoms	40 (71.4%)	27 (71.1%)	13 (72.2%)
Cognitive symptoms	7 (12.5%)	5 (13.2%)	2 (11.1%)
Headache	10 (17.9%)	9 (23.7%)	1 (5.6%)
Cerebral symptoms	8 (14.3%)	8 (21.1%)	0 (0.0%)
Pain	21 (37.5%)	15 (39.5%)	6 (33.3%)
Brainstem symptoms	35 (62.5%)	23 (60.5%)	12 (66.7%)

**Figure 2:**
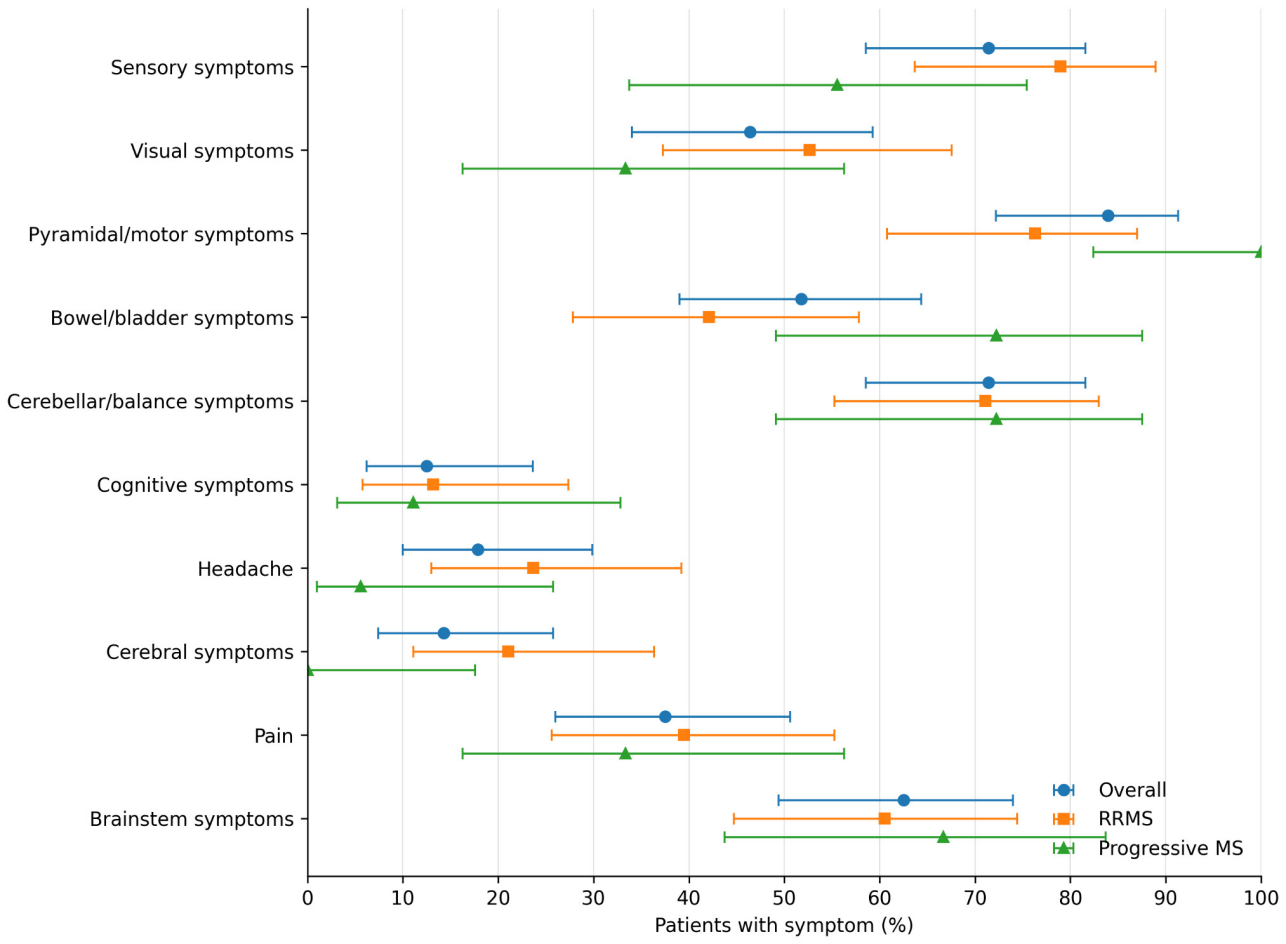
Clinical symptom profile by MS phenotype group. Points indicate observed percentages and error bars indicate 95% Wilson confidence intervals.

MRI data were available for all 56 patients for brain, spinal cord, and optic nerve assessment. The most common MRI lesion locations were periventricular (94.6%), juxtacortical (87.5%), infratentorial (75.0%), optic nerve (50.0%), and spinal cord (82.1%; Table 3).

**Table 3:** Current MRI lesion localization by MS phenotype group.

MRI lesion location	Overall (*n* = 56)	RRMS (*n* = 38)	Progressive MS (*n* = 18)
Periventricular lesions	53 (94.6%)	35 (92.1%)	18 (100.0%)
Infratentorial lesions	42 (75.0%)	24 (63.2%)	18 (100.0%)
Spinal cord lesions	46 (82.1%)	28 (73.7%)	18 (100.0%)
Juxtacortical lesions	49 (87.5%)	33 (86.8%)	16 (88.9%)
Optic nerve lesions	28 (50.0%)	17 (44.7%)	11 (61.1%)

### 3.3 EDSS outcomes and five-year follow-up

Baseline EDSS was significantly higher in the progressive MS group than in RRMS (median 3.75 vs. 2.00; Mann–Whitney *U* = 133.5; *p* < 0.001). Among patients with available five-year follow-up EDSS data, EDSS after five years was also significantly higher in progressive MS than in RRMS (median 6.00 vs. 3.00; *U* = 62.0; exact *p* < 0.001; Figure 3). However, EDSS delta did not differ significantly between RRMS and progressive MS (median 1.00 vs. 0.50; *U* = 144.0; exact *p* = 0.377; Table 4).

**Figure 3:**
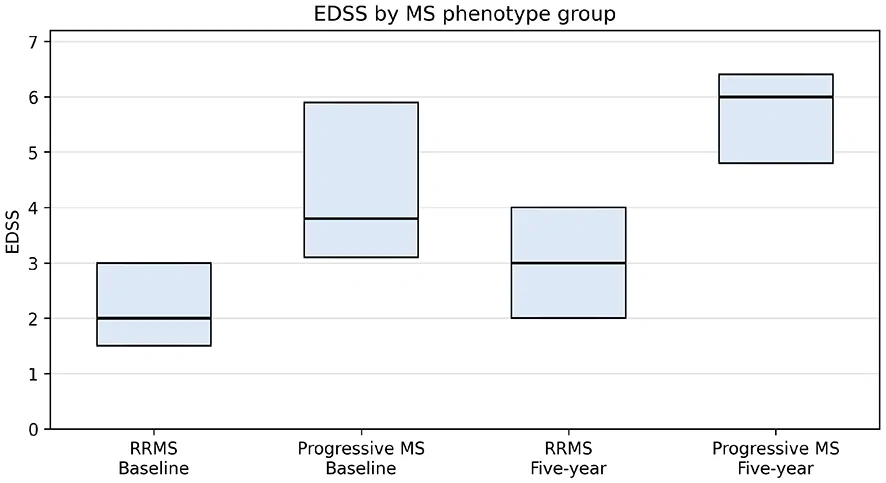
EDSS distribution summarized by median and interquartile range in RRMS and progressive MS at baseline and five-year follow-up. Baseline: RRMS *n* = 38 and progressive MS *n* = 18; five-year follow-up: RRMS *n* = 25 and progressive MS *n* = 14. Boxes represent the interquartile range and the horizontal line represents the median.

**Table 4:** Key EDSS comparisons by MS phenotype group.

Outcome	Groups compared	Statistical test	Result	Interpretation
Baseline EDSS	RRMS vs. progressive MS	Mann–Whitney *U*	*U* = 133.5; *p* < 0.001	Progressive MS had higher baseline disability.
Five-year EDSS (2 groups)	RRMS (*n* = 25) vs. progressive MS (*n* = 14)	Mann–Whitney *U*	*U* = 62.0; exact *p* < 0.001	Progressive MS had higher five-year EDSS.
EDSS delta	RRMS (*n* = 25) vs. progressive MS (*n* = 14)	Mann–Whitney *U*	*U* = 144.0; exact *p* = 0.377	No significant difference in EDSS change.
Five-year EDSS (3 groups)	RRMS vs. PPMS vs. SPMS	Kruskal–Wallis	H(2) = 11.274; *p* = 0.004	Overall difference across phenotypes; SPMS > RRMS after Bonferroni correction (adjusted *p* = 0.007).

Baseline and five-year EDSS scores were higher in patients with progressive MS than in RRMS, whereas the change in EDSS over five years did not differ significantly between groups. However, because the progressive MS group had substantially higher baseline EDSS, the non-significant difference in EDSS change should not be interpreted as evidence of equivalent disability progression between phenotypes. Five-year EDSS data were available for 39 patients: 25 of 38 patients with RRMS (65.8%) and 14 of 18 patients with progressive MS (77.8%). Of the 17 patients without five-year EDSS data, 16 had an age-derived disease duration of less than five years, and missingness was significantly associated with disease duration of less than five years (Fisher's exact *p* < 0.001). In the sensitivity analysis excluding patients with age-derived disease duration of less than five years, 34 patients remained for the baseline comparison (21 RRMS and 13 progressive MS), and 33 had five-year EDSS data (20 RRMS and 13 progressive MS). Baseline EDSS remained significantly higher in progressive MS than in RRMS [median 4.0 (IQR 3.0–6.0) vs. 2.0 (IQR 2.0–4.0); Mann–Whitney *U* = 55.0; exact *p* = 0.003]. Five-year EDSS also remained significantly higher in progressive MS [median 6.0 (IQR 4.0–6.3) vs. 3.0 (IQR 2.1–4.0); *U* = 50.5; exact *p* = 0.002], whereas EDSS change did not differ significantly between groups [median 1.0 (IQR 0.0–1.8) vs. 0.5 (IQR 0.0–1.5); *U* = 112.0; exact *p* = 0.524].

### 3.4 Initial and current treatment patterns

Initial and current treatment patterns differed significantly according to MS phenotype. In analyses restricted to active DMT users, initial anti-CD20 therapy was used in 19.4% of RRMS patients, 88.9% of PPMS patients, and 42.9% of SPMS patients (Fisher–Freeman–Halton exact *p* < 0.001; Cramer's *V* = 0.547). In the initial DMT intensity analysis, which included all patients, platform/moderate DMT was most common in RRMS, whereas high-efficacy DMT was more common in PPMS and SPMS (Fisher–Freeman–Halton exact *p* < 0.001; Cramer's *V* = 0.416). The distribution of current individual DMTs is shown in Table 5.

**Table 5:** Current disease-modifying therapy distribution.

Therapy	*n*	% of cohort
No DMT	4	7.1%
Glatiramer acetate	3	5.4%
Dimethyl fumarate	9	16.1%
Teriflunomide	3	5.4%
Cladribine	2	3.6%
Ofatumumab	17	30.4%
Ocrelizumab	11	19.6%
Ponesimod	2	3.6%
Interferon beta-1a	2	3.6%
Prednisolone	1	1.8%
Diroximel fumarate	2	3.6%

Current anti-CD20 therapy was used in 38.9% of active-DMT-treated RRMS patients, 100.0% of PPMS patients, and 83.3% of active-DMT-treated SPMS patients (Fisher–Freeman–Halton exact *p* < 0.001; Cramer's *V* = 0.506). Current treatment intensity also differed according to MS phenotype, with all PPMS patients receiving high-efficacy DMT and RRMS patients most often receiving platform/moderate DMT (Fisher–Freeman–Halton exact *p* < 0.001; Cramer's *V* = 0.415; Figure 4; Table 6).

**Figure 4:**
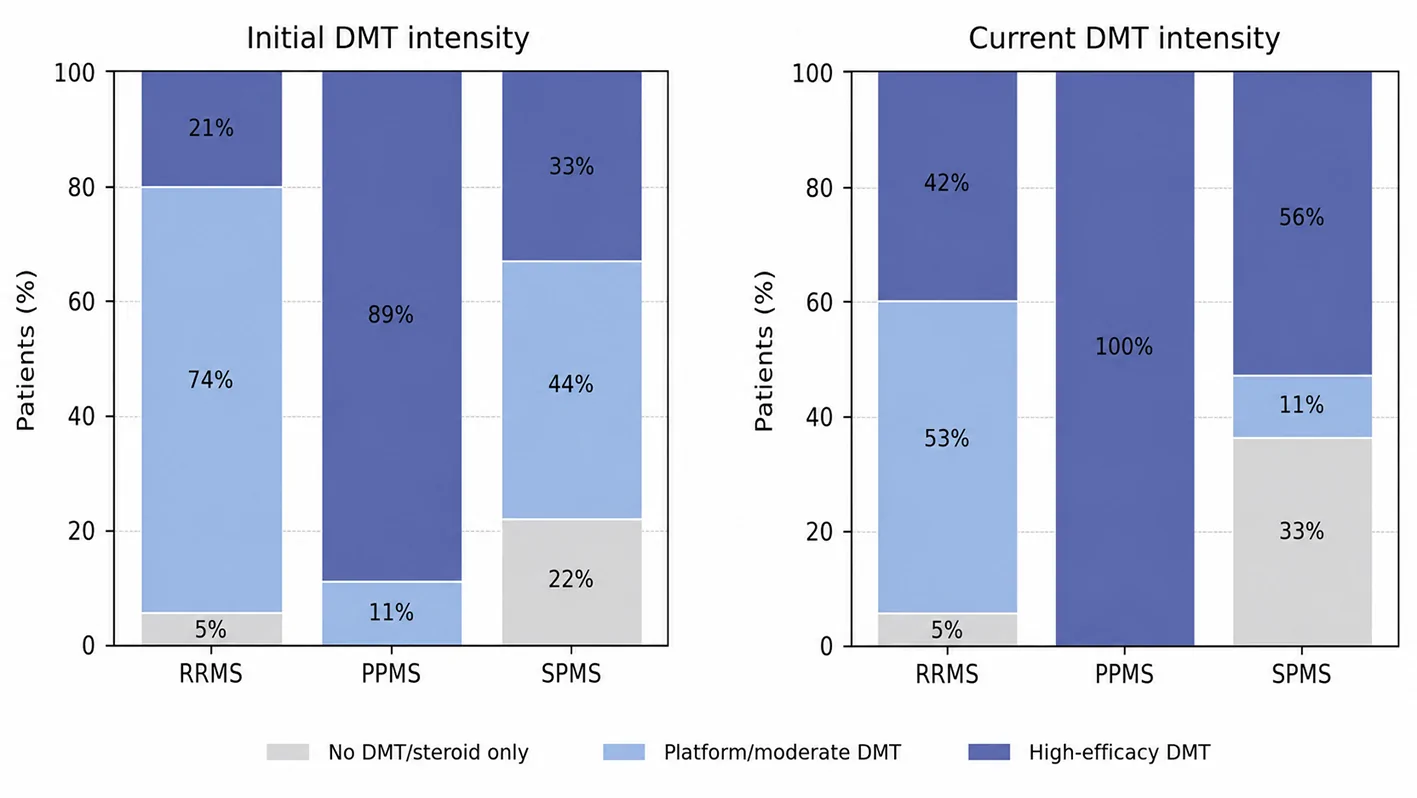
Initial and current DMT intensity distributions by MS phenotype. Percentages are calculated within each phenotype group.

**Table 6:** Initial and current DMT intensity groups by MS phenotype.

Time point	MS phenotype	No DMT/steroid only	Platform/moderate DMT	High-efficacy DMT	Total
Initial	RRMS	2 (5.3%)	28 (73.7%)	8 (21.1%)	38
Initial	PPMS	0 (0.0%)	1 (11.1%)	8 (88.9%)	9
Initial	SPMS	2 (22.2%)	4 (44.4%)	3 (33.3%)	9
Current	RRMS	2 (5.3%)	20 (52.6%)	16 (42.1%)	38
Current	PPMS	0 (0.0%)	0 (0.0%)	9 (100.0%)	9
Current	SPMS	3 (33.3%)	1 (11.1%)	5 (55.6%)	9

Comparison of initial and current treatment categories showed changes in treatment intensity over time. Among the 33 patients initially classified as receiving platform/moderate DMT, 21 (63.6%) remained in the platform/moderate group, 11 (33.3%) were receiving high-efficacy DMT at the current assessment, and 1 (3.0%) was classified as no DMT/steroid only. All 19 patients initially receiving high-efficacy DMT remained in the high-efficacy treatment category at the current assessment (Figure 5).

**Figure 5:**
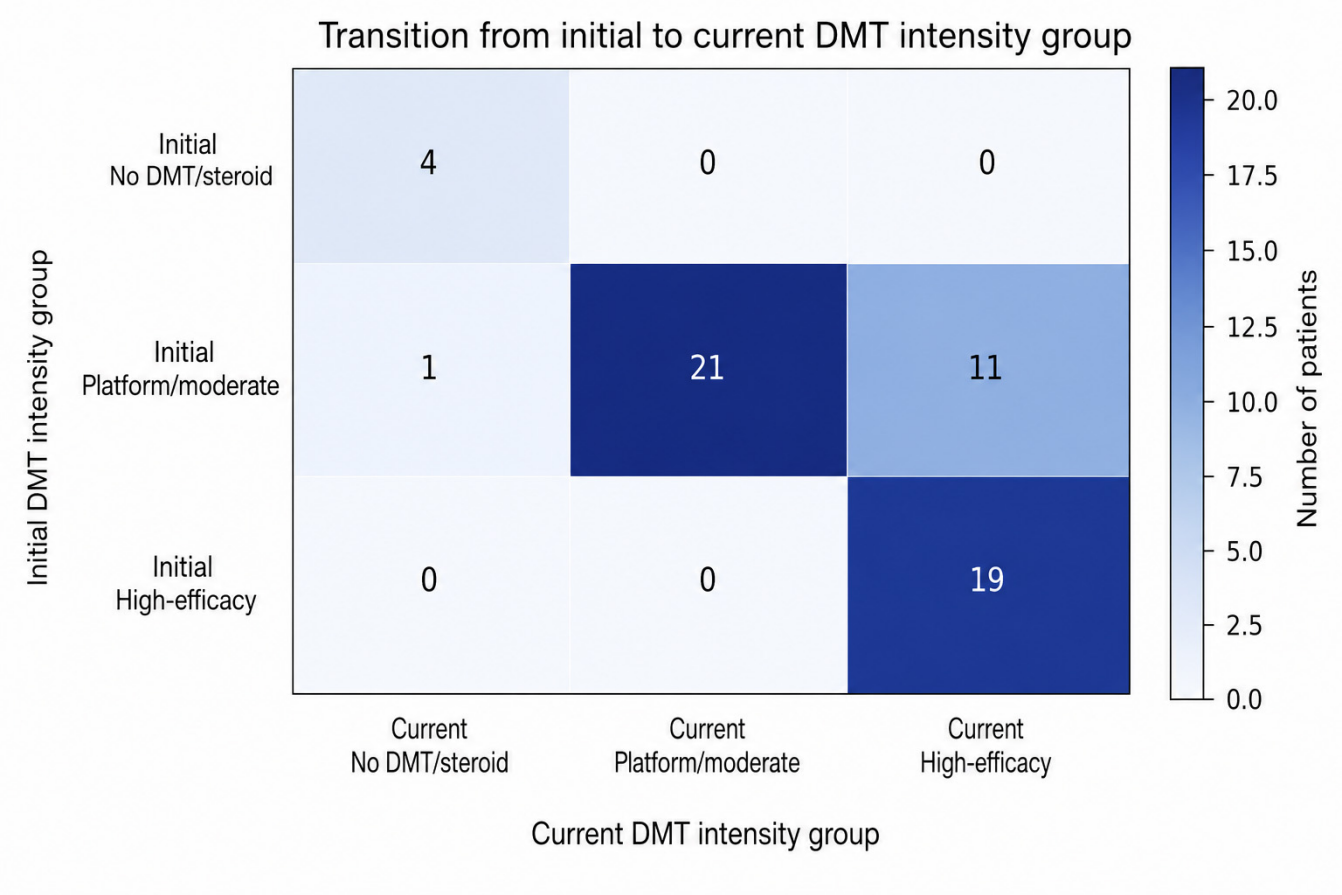
Transition from initial to current DMT intensity group. Cell values represent patient counts.

### 3.5 Treatment groups and disability measures

Patients receiving initial anti-CD20 therapy had higher baseline EDSS than those receiving other active DMTs [median 3.5 (IQR 2.0–6.0) vs. 2.0 (IQR 1.5–3.1); Mann–Whitney *U* = 185.0; *p* = 0.019]. Baseline EDSS showed no statistically significant difference across initial DMT intensity groups [no DMT/steroid only: median 4.5 (IQR 2.4–5.9), platform/moderate DMT: median 2.0 (IQR 1.5–3.3), high-efficacy DMT: median 3.5 (IQR 2.0–6.0); Kruskal–Wallis H(2) = 5.733; *p* = 0.057].

Patients receiving current anti-CD20 therapy also had higher baseline EDSS than those receiving other active DMTs [median 3.5 (IQR 2.0–5.0) vs. 2.0 (IQR 1.5–2.5); Mann–Whitney *U* = 179.0; *p* = 0.006]. Among patients with available five-year EDSS data, current anti-CD20 therapy was also associated with higher five-year EDSS compared with other active DMTs [median 5.0 (IQR 3.5–6.1) vs. 2.5 (IQR 2.0–3.8); Mann–Whitney *U* = 60.0; exact *p* = 0.002]. Five-year EDSS differed across current DMT intensity groups, with the lowest median EDSS in the platform/moderate DMT group and higher values in the high-efficacy and no DMT/steroid-only groups [2.5 (IQR 2.0–3.9), 4.5 (IQR 3.5–6.0), and 6.0 (IQR 3.8–6.4), respectively; Kruskal–Wallis H(2) = 8.908; *p* = 0.012]. After Bonferroni correction, five-year EDSS was significantly higher in the high-efficacy DMT group than in the platform/moderate DMT group (adjusted *p* = 0.027; Table 7).

**Table 7:** Treatment-group associations with EDSS outcomes.

Analysis	Test	Result	Interpretation
Baseline EDSS by initial anti-CD20 status	Mann–Whitney *U*	*U* = 185.0; *p* = 0.019	Initial anti-CD20 group had higher baseline EDSS.
Baseline EDSS by initial DMT intensity	Kruskal–Wallis	H(2) = 5.733; *p* = 0.057	No statistically significant overall difference in baseline EDSS across initial DMT intensity groups.
Baseline EDSS by current anti-CD20 status	Mann–Whitney *U*	*U* = 179.0; *p* = 0.006	Current anti-CD20 group had higher baseline EDSS.
Five-year EDSS by current anti-CD20 status	Mann–Whitney *U*	*U* = 60.0; exact *p* = 0.002	Current anti-CD20 group had higher five-year EDSS.
Five-year EDSS by current DMT intensity	Kruskal–Wallis	H(2) = 8.908; *p* = 0.012; high-efficacy vs. platform/moderate adjusted *p* = 0.027	High-efficacy DMT group had higher five-year EDSS than platform/moderate DMT group.

Patients receiving anti-CD20 therapy or high-efficacy DMTs had higher observed baseline and five-year EDSS scores than patients receiving other therapies. These comparisons are descriptive and are likely influenced by confounding by indication, because patients with progressive disease and greater baseline disability were more likely to receive higher-efficacy treatment.

## 4 Discussion

The principal contribution of this study is not the identification of a previously unrecognized LOMS phenotype, but the contemporary replication and extension of established observations in a Latvian clinical setting. Nearly five decades ago, Cazzullo et al. (10) reported frequent motor onset, progressive disease, and greater disability in patients with later-onset MS. The present findings are broadly consistent with that historical description: progressive phenotypes were associated with greater pyramidal and bowel/bladder symptom burden and with higher baseline and five-year EDSS. However, the present study extends the historical evidence by applying a symptom-onset threshold of ≥50 years and by integrating contemporary MRI lesion localization, longitudinal disability assessment, and initial-to-current DMT patterns, including anti-CD20 and other high-efficacy therapies. Thus, consistency with earlier research should be interpreted as external replication under substantially different diagnostic and therapeutic conditions rather than as a claim of discovering a new clinical phenotype.

The Latvian setting is important because published full-length data on LOMS from this population remain scarce. This single-center cohort cannot be considered nationally representative; nevertheless, it provides a detailed account of LOMS in contemporary Latvian tertiary practice and adds evidence from a geographically underrepresented European setting. Internationally, such country-specific cohorts contribute to evaluating whether established LOMS characteristics are reproducible across different populations and healthcare systems. They also provide granular information on MRI findings and real-world treatment selection that may not be available in older studies or large registry analyses. These data may therefore support future multicenter comparisons, pooled analyses, and the development of more generalizable evidence regarding the diagnosis, disability burden, and treatment of older-onset MS.

However, the lack of a significant difference in unadjusted EDSS change should be interpreted in the context of substantial baseline disability differences and incomplete five-year follow-up data. Late-onset multiple sclerosis represents a clinically heterogeneous subgroup of MS in which older age at symptom onset may overlap with progressive disease course, higher baseline disability, diagnostic complexity, and real-world treatment-selection challenges. Recent consensus recommendations on MS in older adults emphasize that aging, immunosenescence, comorbidities, and limited trial evidence complicate both diagnostic interpretation and DMT decision-making in this population (21). In the present retrospective single-center cohort, the interpretation of disability outcomes was strongly influenced by baseline differences between phenotypes. Patients with progressive MS had higher EDSS at both baseline and five-year follow-up; however, EDSS change over five years did not differ significantly between RRMS and progressive MS. This suggests that the higher disability observed after five years was largely related to disability already present at the beginning of observation rather than a clearly faster rate of disability accumulation during the follow-up period.

These disability findings can also be viewed alongside large registry-based studies. In the Italian MS Register, Lorefice et al. identified four longitudinal EDSS trajectories among 16,159 patients with relapsing-remitting MS, including 1,012 with LOMS; LOMS was associated with a greater probability of belonging to the more severe disability trajectories (22). In a separate Italian MS Register analysis of pediatric-, adult-, and late-onset MS, Simone et al. found greater disability accumulation with increasing age at onset and a pronounced effect of progression independent of relapse activity in LOMS (23). International analyses from the Big Multiple Sclerosis Data Network have likewise demonstrated marked heterogeneity in long-term disability trajectories in progressive MS, with distinct mild, moderate, and severe courses rather than a uniform progression rate (24). These large-scale observations are broadly compatible with the greater absolute disability burden observed in our progressive LOMS group, while also emphasizing that our two-time-point, single-center design cannot characterize individual disability trajectories or progression mechanisms with the same granularity as longitudinal registry studies.

The demographic and clinical characteristics of the present cohort were broadly consistent with previous studies of LOMS. Naseri et al. (1) reported that LOMS accounts for approximately 5% of MS cases and remains more common in women than men, although the female predominance appears less pronounced than in younger-onset MS. In the present cohort, 67.9% of patients were female. The proportion of progressive phenotypes was also consistent with previous evidence showing that progressive disease courses are more common in LOMS than in adult-onset MS (3, 11–13). Similarly, Ozakbas et al. (16) reported higher EDSS scores in LOMS compared with adult- and early-onset MS groups at admission, during early follow-up, and at current assessment, supporting the concept that older-onset MS is associated with a greater disability burden. These findings are also consistent with recent data comparing younger- and older-onset MS, which showed that older-onset MS may have lower inflammatory activity but still be associated with substantial disability burden (20). The nationwide Swedish study by Mouresan et al. also showed that patients with LOMS had a higher risk of reaching EDSS scores of 4 and 6 compared with those with adult-onset MS, despite the availability of modern DMTs (3).

The clinical symptom profile in this cohort further supports the relevance of motor and long-tract involvement in LOMS. Motor or pyramidal manifestations were frequent overall and were present in all patients with progressive MS, while bowel and bladder dysfunction were also more common in progressive phenotypes. These findings are clinically plausible because progressive MS and spinal cord involvement are often associated with gait impairment and sphincter symptoms. Motor or pyramidal manifestations have also been repeatedly described as common clinical features of LOMS in previous studies (1, 13, 14). Therefore, the greater symptom burden observed in progressive MS in the present cohort is consistent with the broader clinical profile reported in older-onset MS populations.

MRI findings demonstrated involvement of several typical MS-related anatomical regions. Periventricular lesions were the most frequent MRI finding, followed by juxtacortical, spinal cord, and infratentorial lesions, while optic nerve involvement was documented in half of the cohort. This broad lesion distribution supports multifocal CNS involvement and is consistent with the diagnostic principle of dissemination in space. Among these localizations, spinal cord involvement is particularly relevant in LOMS because it may contribute to pyramidal symptoms, gait impairment and bowel/bladder dysfunction. Infratentorial involvement may also be relevant for brainstem and cerebellar manifestations, while optic nerve involvement indicates that visual pathway involvement remains part of the clinical-radiological spectrum even in older-onset disease. Although the frequency of spinal cord lesions in the present cohort was higher than the pooled estimate reported in a radiological systematic review of LOMS, the direction of the finding is consistent with published evidence that spinal cord involvement is common in LOMS (2). The high frequency observed in this study may partly reflect the tertiary hospital setting, where patients with more complex or clinically pronounced neurological symptoms are more likely to be evaluated.

Diagnostic interpretation of MRI findings in LOMS requires particular caution. Older patients more frequently have vascular, degenerative, metabolic, or inflammatory disorders that can mimic MS clinically or radiologically (4, 5). Therefore, typical lesion localization should not be interpreted in isolation but should be integrated with the clinical presentation, spinal cord imaging, cerebrospinal fluid oligoclonal bands, and, where available, more specific MRI markers such as the central vein sign (6). These considerations support comprehensive MRI assessment, including brain, spinal cord, and optic pathway evaluation, when investigating suspected MS in older patients.

Treatment analyses showed that anti-CD20 and other high-efficacy DMTs were used more frequently in patients with progressive phenotypes and higher baseline EDSS. This pattern should not be interpreted as evidence that these therapies were associated with worse outcomes. In routine observational clinical practice, treatment allocation is not random; clinicians may preferentially prescribe higher-efficacy therapies to patients with more severe disease, higher disability, progressive phenotypes, or evidence of inflammatory activity. This interpretation is supported by registry data showing that EDSS level, relapse activity, MRI lesion burden, disease duration, age, and sex influence the choice of high-efficacy DMT and treatment escalation in real-world MS practice (15). The observed disability differences therefore most likely reflect confounding by indication rather than a negative treatment effect.

The inclusion of both initial and current treatment data strengthens this interpretation. Patients receiving initial anti-CD20 therapy already had higher baseline EDSS than those receiving other active DMTs, and baseline EDSS differed overall across initial DMT intensity groups. Thus, disability differences existed before the current treatment classification and should not be attributed solely to current therapy. The comparison between initial and current DMT intensity groups suggested escalation in a subset of patients, particularly from platform/moderate DMT to high-efficacy DMT. This pattern is consistent with the broader real-world MS treatment literature, in which escalation from low- or moderate-efficacy therapy to high-efficacy DMT has been compared with early initiation of highly effective therapy in RRMS cohorts (19). However, because treatment data were analyzed retrospectively and not fully time-aligned with disability outcomes, these results are better suited to describing treatment patterns than to evaluating causal treatment effectiveness. A causal analysis would require exact treatment start dates, relapse and MRI activity over time, therapy-switch dates, treatment adherence, adverse events, and preferably multivariable or propensity-score methods.

This study has several strengths. It focuses on a clinically important but underrepresented MS subgroup and uses real-world data from a tertiary hospital setting. The analysis included clinical symptoms, MRI lesion localization, EDSS-based disability outcomes, and both initial and current DMT classifications. The study also explicitly addressed the missing five-year EDSS data and interpreted treatment-related findings cautiously given the observational study design and the possibility of confounding by indication.

This study has several limitations. Its retrospective single-center design and modest sample size, particularly within the PPMS and SPMS subgroups, limited statistical power and adjustment for potential confounders. Because the study period spanned revisions of the McDonald diagnostic criteria, patients were diagnosed according to the criteria applicable at the time of diagnosis, which may have introduced some diagnostic heterogeneity. Because the study did not include an adult-onset MS comparator group, it cannot determine whether the observed clinical and disability patterns are specific to late-onset disease or primarily reflect differences between relapsing and progressive MS phenotypes. The study should therefore be interpreted as a detailed characterization of contemporary LOMS rather than as a direct comparison between late- and younger-onset MS. MRI data were extracted from routine neuroradiology reports rather than from centralized image review. Five-year EDSS data were unavailable for some patients, mainly because of insufficient follow-up duration. Treatment exposure was not fully time-aligned with disability assessments, and relapse activity, MRI activity over time, treatment adherence, therapy-switch dates, and adverse events were not systematically evaluated. Finally, multiple exploratory comparisons were performed; therefore, borderline associations should be interpreted cautiously.

## 5 Conclusion

This single-center retrospective study provides a contemporary characterization of LOMS in a Latvian tertiary care cohort. The results confirm that historically recognized motor, sphincter, and disability burdens remain evident among patients with progressive LOMS phenotypes, while extending earlier evidence through the integration of MRI lesion localization, five-year EDSS outcomes, and modern DMT patterns. The study does not establish a new LOMS phenotype or comparative treatment effectiveness. Its contribution lies in providing detailed real-world evidence from a setting with limited published full-length LOMS data and in demonstrating the relevance of established clinical observations under contemporary diagnostic and therapeutic conditions. These findings support the inclusion of geographically diverse cohorts in future multicenter and pooled studies of older-onset MS.

## Data Availability

The individual-level clinical data analyzed in this study are not publicly available because they are subject to patient confidentiality and institutional restrictions. Requests to access anonymized data should be directed to the corresponding author at trundik2002@gmail.com and will be considered subject to applicable ethics and institutional approvals.
