# Supplementary material for: Clinical characteristics, disability outcomes, and disease-modifying therapy patterns in late-onset multiple sclerosis: a single-center retrospective cohort study
Source: Front Neurol. 2026 Oct 21;17:1966162. doi: 10.3389/fneur.2026.1966162 (PMC13730846; doi:10.3389/fneur.2026.1966162)
Supplement: Supplementary file 1 [file Table_1.docx]

**Supplementary Material**

**STROBE and RECORD Reporting Checklist**

*Clinical Characteristics, Disability Outcomes, and Disease-Modifying Therapy Patterns in Late-Onset Multiple Sclerosis: A Single-Center Retrospective Cohort Study*

This checklist maps the revised manuscript to the STROBE recommendations for cohort studies and the RECORD extension for observational studies using routinely collected health data. “N/A” indicates an item that does not apply to this study.

**A. STROBE checklist for cohort studies**

| **Item** | **Reporting requirement** | **Location in revised manuscript** | **Status / note** |
| --- | --- | --- | --- |
| 1a | Identify the observational cohort design in the title or abstract. | Title; Abstract (p. 1) | Addressed |
| 1b | Provide a balanced abstract summary of methods and findings. | Abstract (p. 1) | Addressed |
| 2 | Explain the scientific background and rationale. | Introduction (pp. 1–2) | Addressed |
| 3 | State the study objectives and any prespecified hypotheses. | Introduction (p. 2) | Addressed; primary and secondary objectives stated |
| 4 | Present key study-design elements early in the manuscript. | Materials and methods, Study design and setting (p. 3) | Addressed |
| 5 | Describe setting, location, and relevant dates. | Study design and setting (p. 3) | Addressed: Riga East University Hospital; 2014–2024 |
| 6a | Describe eligibility criteria and sources/methods of participant selection and follow-up. | Study population (p. 3); Figure 1 | Addressed |
| 6b | For matched studies, report matching criteria and group numbers. | N/A | Not a matched cohort |
| 7 | Define outcomes, predictors/exposures, potential confounders/effect modifiers, and diagnostic criteria as applicable. | Study population; Variables and definitions; Treatment classification; Outcomes (pp. 3–4) | Addressed for variables analyzed |
| 8 | Give data sources and methods of measurement/assessment for variables. | Study design and setting; Variables and definitions (p. 3) | Addressed; routine medical records and neuroradiology reports |
| 9 | Describe efforts to address potential sources of bias. | Methods (missing follow-up sensitivity analysis); Discussion limitations (pp. 4, 8) | Addressed |
| 10 | Explain how study size was determined. | Study population (p. 3) | Addressed in manuscript: eligible cases available in source dataset |
| 11 | Explain how quantitative variables were handled and any groupings used. | Variables and definitions; Treatment classification; Statistical analysis (pp. 3–4) | Addressed |
| 12a | Describe statistical methods, including methods used for confounding if applicable. | Statistical analysis (p. 4) | Addressed; no multivariable confounder-adjusted model was performed |
| 12b | Describe subgroup/interaction analyses where applicable. | Statistical analysis; Results (pp. 4–6) | Addressed for phenotype and treatment-group comparisons |
| 12c | Explain how missing data were handled. | Outcomes; Study population; Results, EDSS follow-up (pp. 3–5) | Addressed: missing values were retained as missing and were not imputed; five-year EDSS availability and missingness were analyzed explicitly |
| 12d | Explain how loss to follow-up was addressed when applicable. | Outcomes; Results, EDSS follow-up (pp. 4–5) | Addressed through availability analysis and sensitivity analysis |
| 12e | Describe sensitivity analyses. | Outcomes (p. 4); Results (p. 5) | Addressed |
| 13a | Report participant numbers at each stage of the study. | Study cohort (p. 4); Figure 1 | Addressed |
| 13b | Give reasons for exclusions/non-participation at each stage. | Study population; Figure 1 (pp. 3–4) | Addressed |
| 13c | Consider a participant flow diagram. | Figure 1 | Addressed |
| 14a | Report participant demographic/clinical characteristics and relevant exposures/confounders. | Table 1; Results (pp. 4–5; Table 1 at end) | Addressed |
| 14b | Indicate numbers with missing data for variables of interest. | Results, EDSS follow-up (p. 5); Table 1 note | Addressed explicitly for five-year EDSS (39 available, 17 missing) |
| 14c | Summarize follow-up time. | Table 1 disease duration; five-year EDSS outcome definition (pp. 4–5) | Addressed for the main longitudinal outcome |
| 15 | Report outcome events or summary measures over time. | Results (pp. 5–6); Tables 1, 4, 7 | Addressed |
| 16a | Report unadjusted estimates and precision; adjusted estimates if applicable. | Results (pp. 5–6); Figure 2; Tables 1–7 | Unadjusted medians/IQRs, proportions, p-values; 95% Wilson CIs in Figure 2 |
| 16b | Report category boundaries when continuous variables are categorized. | N/A / Methods | No principal continuous outcome was categorized for the main phenotype comparison |
| 16c | Translate relative risk into absolute risk when relevant. | N/A | Not a risk-estimation study |
| 17 | Report other analyses, including subgroup and sensitivity analyses. | Results (pp. 5–6) | Addressed |
| 18 | Summarize key results with reference to objectives. | Discussion (pp. 6–8) | Addressed |
| 19 | Discuss limitations, bias, and imprecision. | Discussion limitations (p. 8) | Addressed |
| 20 | Provide a cautious overall interpretation considering objectives, limitations, multiplicity, and prior evidence. | Discussion (pp. 6–8) | Addressed |
| 21 | Discuss generalizability/external validity. | Discussion (pp. 6–8); Conclusion (p. 9) | Addressed |
| 22 | Report funding source and role of funders. | Funding (p. 9) | Addressed: no external funding |

**B. RECORD extension for routinely collected health data**

| **Item** | **Reporting requirement** | **Location in revised manuscript** | **Status / note** |
| --- | --- | --- | --- |
| 1.1 | Specify the type of routinely collected health data in the title or abstract; name the database if appropriate. | Abstract (p. 1): retrospective cohort based on medical records from Riga East University Hospital | Addressed |
| 1.2 | Report geographic region and study time frame in the title/abstract when applicable. | Title/Abstract (p. 1); Methods (p. 3) | Latvia reported in abstract; 2014–2024 reported in Methods |
| 1.3 | State database linkage in title/abstract if linkage was performed. | N/A | No linkage is described in the study |
| 6.1 | Describe in detail how the study population was selected from the routine-data source. | Study population (p. 3); Figure 1 | Addressed: 518 screened → 59 LOMS → 56 analytic cohort |
| 6.2 | Reference/describe validation of codes or algorithms used to select the population, if such algorithms were used. | N/A | No administrative-code selection algorithm is reported |
| 6.3 | If databases were linked, use a flow diagram or display to show the linkage process. | N/A | No database linkage reported |
| 7.1 | Provide the codes/algorithms used to classify exposures, outcomes, confounders, or effect modifiers. | Variables and definitions; Treatment classification (p. 3) | Clinical variables were defined directly in the manuscript; no external administrative coding algorithm reported |
| 12.1 | Describe investigators’ access to the source database population. | Study population (p. 3) | Addressed: the deduplicated source dataset comprised 518 unique patients and was screened for LOMS eligibility |
| 12.2 | Describe data-cleaning methods used. | Study population (p. 3) | Addressed: duplicate records were identified using unique patient identifiers and removed before creation of the 518-patient source dataset; missing values were retained as missing and were not imputed |
| 12.3 | Describe person-level/institution-level linkage methods and linkage quality if multiple databases were linked. | N/A | No linkage reported |
| 13.1 | Describe selection/filtering of included persons, including filtering for data availability/quality; a flow diagram may be used. | Study population (p. 3); Figure 1; Results (p. 4) | Addressed |
| 19.1 | Discuss limitations arising because routine clinical data were not collected specifically for the research question. | Discussion limitations (p. 8) | Addressed: routine MRI reports, missing follow-up, unmeasured longitudinal activity/adherence/switch data, confounding by indication |
| 22.1 | State how supplemental information, protocol, raw data, or analysis code can be accessed. | Data availability statement (p. 9) | Addressed: patient-level data are not publicly available because of confidentiality and institutional restrictions; any access request is subject to applicable ethics and institutional approvals |

Submission note: The manuscript reports deduplication of source records using unique patient identifiers, retention of missing values without imputation, and restrictions on public access to patient-level data because of ethical, confidentiality, and institutional requirements. No database linkage was performed.
